# Comparison between esomeprazole 20 mg Vs 40 mg as stress ulcer prophylaxis (SUP) in critically ill patients: A retrospective cohort study

**DOI:** 10.1002/prp2.624

**Published:** 2020-07-23

**Authors:** Khalid Al Sulaiman, Kholoud Al Aamer, Alaa Al Harthi, Saud Jaser, Abdulrahman Al Anazi, Sultan Al Subaie, Ramesh Vishwakarma

**Affiliations:** ^1^ Pharmaceutical Care Department‐King Abdulaziz Medical City‐MNGHA King Abdullah International Medical Research Center/King Saud bin Abdulaziz University for Health Sciences Riyadh Saudi Arabia; ^2^ Department of Biostatistics and Bioinformatics‐King Abdulaziz Medical City‐MNGHA King Abdullah International Medical Research Center King Saud bin Abdulaziz University for Health Sciences Riyadh Saudi Arabia

**Keywords:** critically ill, esomeprazole, ICU, PPI, stress ulcer prophylaxis

## Abstract

Critically ill patients admitted to intensive care units (ICUs) are at high risk of developing upper gastrointestinal bleeding due to GI stress ulceration (SU). The major independent risk factors for the development of GI bleeding in the ICUs include mechanical ventilation (MV) and coagulopathy. There is no enough evidence regarding the most appropriate dosing of esomeprazole as stress ulcer prophylaxis (SUP) in critically ill patients. This is a retrospective cohort study conducted at King Abdulaziz Medical City‐Riyadh between January and December 2018 to determine the efficacy and safety of two different regimens of esomeprazole (20 vs 40 mg) as SUP in critically ill patients with major risk factors of GI stress ulceration. A total of 1864 patients were reviewed, 387 patients meeting inclusion criteria were enrolled. The propensity score was used to adjust for clinically and statistically relevant variables. We considered a *P* value of <.05 as statistically significant. 49 patients (12.6%) had received Esomeprazole 20 mg during the study period. Compared with Esomeprazole 20 mg, Esomeprazole 40 mg was not superior in GI bleeding prevention (aOR 2.611, 95% CI 0.343‐20.247, *P* = .356). In addition, neither ICU C. difficle, ICU mortality within 30 days, ICU LOS, hospital LOS, ICU re‐admission within 6 months, RBCs transfusion, nor platelets transfusion requirements were significant. On the other hand, Esomeprazole 40 mg was statistically associated with Enterobacteriaceae, Pneumonia, and longer MV duration.

**Abbreviations**
GI: gastrointestinal
GU: gastric ulcer
H2RAs: H2 receptor antagonists
ICUs: intensive care units
MV: mechanical ventilation
SU: stress ulceration
SUP: stress ulcer prophylaxis

## 1 INTRODUCTION

Critically ill patients admitted to intensive care units (ICUs) are at high risk of developing upper gastrointestinal bleeding (UGIB) due to GI stress ulceration (SU) leading to several consequences.^1^ No specific definition for SU, nevertheless can be defined as an acute, erosive, inflammatory insult that causes mucosal injury which varies from superficial ulcers to deep bleeding lesions that occur after the first 24 hours of hospitalization.^1^, ^2^ The prevalence of gastrointestinal (GI) bleeding is different between studies because of mixed populations.^3^ GI bleeding secondary to SU after mechanical ventilation in ICU Patients have been noted, as studies reported an incidence of SU and subepithelial hemorrhage within 24 hours of admission in ICU patients.^3^ The major risk factors for the development of GI bleeding in the ICUs include mechanical ventilation, coagulopathy, and hepatic or kidney failure.^4^ Observational studies showed that proton pump inhibitors (PPIs) are the most commonly used prophylactic agents in the ICU.^5^ A meta‐analysis provides moderate quality evidence for clinicians and guideline groups suggesting that PPIs, when compared to H2RAs, lower the risk of clinically important and overt GI bleeding among critically ill patients, without increasing the risk of pneumonia and mortality, or ICU length of stay.^5^ A study of NSAID‐associated GU healing was not statistically different when comparing different esomeprazole dosing regimen (20 vs 40 mg esomeprazole) after 8 weeks.^6^ Another study by Plein et al compared 20 vs 40 mg pantoprazole, found that 20 mg pantoprazole was safe and effective in maintaining patients with healed reflux esophagitis in remission. Additionally, 20 mg of pantoprazole provides good long‐term therapeutic efficacy at less drug exposure and lower costs.^7^

There is no enough evidence regarding the most appropriate dosing of esomeprazole as stress ulcer prophylaxis in critically ill patients. This study aims to investigating the efficacy and safety for different regimens of esomeprazole (20 mg vs 40 mg) as stress ulcer prophylaxis in critically ill patients with major risk factors for stress ulceration.

## 2 METHODS

### 2.1 Study design

A retrospective cohort study of adult ICU patients at King Abdulaziz Medical City‐Riyadh who received esomeprazole as stress ulcer prophylaxis (SUP) between January 1st, 2018 and December 31st, 2018 to determine the efficacy and safety of different regimen of esomeprazole (20 mg vs 40 mg) as SUP in critically ill patients with major risk factors. A total of 1864 patients were reviewed to screen patients for inclusion into the study, 387 patients meeting inclusion/exclusion criteria were enrolled. Patients were divided into two groups based on esomeprazole dosing (20 mg vs 40 mg). The study was approved by King Abdullah International Medical Research Center Institutional Review Board, Riyadh, Saudi Arabia.

### 2.2 Setting

This study was conducted in the adult medical, surgical, trauma, and burn ICUs at King Abdulaziz Medical City (KAMC)‐National Guard Health Affairs (NGHA), which is a tertiary‐care academic referral hospital in Riyadh, Saudi Arabia. The ICU admits medical, surgical, trauma, burn patients, and operates as a closed unit with 24/7 onsite coverage by critical care board‐certified intensivists. Clinical pharmacists’ specialists, respiratory therapists and nurses are a part of the daily multidisciplinary rounds. The nurse‐to‐patient ratio in the unit is approximately 1:1.2.^8^

### 2.3 Data collection

Demographic and clinical data including age, gender, weight, body mass index (BMI), associated co‐morbidities, laboratory baseline within 24 hours of ICU admission, Glasgow Coma Scale (GCS), Vasoactive Inotropic Score (VIS), Acute Physiology and Chronic Health Evaluation (APACHE II) score, Sequential Organ Failure Assessment (SOFA) score, and Nutrition Risk in Critically ill (NUTRIC) score were recorded for eligible patients on the first day. In addition, mechanical ventilation, endoscopy, RBCs/platelets transfusion, plasma frozen plasma (FFP) transfusion, previous C. difficle (Within 6 months of ICU admission), ICU C. difficle, pneumonia, blood, and urinary cultures were reviewed and recorded.

### 2.4 Eligibility criteria

Patients were enrolled in the study if they were critically ill aged 16 y/o or older with at least one major risk factors of stress ulceration (i.e. requiring MV > 24 hours and/or coagulopathy (INR>1.5 or platelets< 50,000/microliter)) and administered esomeprazole (either parenteral or enteral) for 48 hours as stress ulcer prophylaxis. Exclusion criteria included a primary diagnosis of GI bleeding within 24 hours of ICU admission, hepatic Failure ( Child B – C), Liver cirrhosis, administration of PPIs exceeding once‐daily dosing, administration of different prophylactic dosing of esomeprazole in sequential (except treatment dose) while in ICU stay, PPI is indicated for an indication other than SUP (i.e. Helicobacter pylorieradication, erosive esophagitis, varices hemorrhage and/or gastroesophageal reflux (GERD)), administration of both H2RA and esomeprazole while in ICU (sequential or concurrent use), ICU length of stay (LOS) <1 day or >60 days and “Do‐Not‐Resuscitate” status within 24 hours of admission.

### 2.5 Availability of data and material

Data are available upon request due to privacy/ethical restrictions.

### 2.6 Outcomes

The primary outcome was GI bleeding, pneumonia, bacteremia, significant urinary tract infection, ICU Clostridium difficile. GI bleeding determined with GI endoscopy and/or receiving PPI as a treatment dose with documentation of GI bleeding. Clostridium difficile confirmed with a positive polymerase chain reaction (PCR). The secondary outcomes were RBCs/platelets transfusion requirement, mechanical ventilation duration, ICU/hospital length of stay and ICU mortality.

### 2.7 Data management and statistical analysis

Collected data were entered in Microsoft Excel after being coded. There were two arms considered in this study, patients who received esomeprazole 20 vs 40 mg as stress ulcer prophylaxis. As expected in an observational study, differences in baseline characteristics between the two treatment groups may exist. To adjust for these differences, a propensity score for the use of Esomeprazole 20 mg was generated with Apache II scores. Baseline characteristics, baseline severity, and outcome variables were compared between the two groups.

Categorical variables were presented as percentages and numerical variables (continuous variables) as mean and standard deviation (SD). The normality assumptions were assessed for all numerical variables using a statistical test (ie Shapiro‐Wilk test) and also using graphical representation (ie histograms and Q‐Q plots). We compared categorical variables using the chi‐square or Fisher exact test, normally distributed numerical variables with the *t*‐test, and other quantitative variables with the Mann‐Whitney *U* test.

Multivariate logistic regression was used to find out the relationship between treatments and the different outcomes considered in this study, adjusting for the generated propensity score.

We assessed model fit using the Hosmer‐Lemeshow goodness‐of‐fit test. Generalized linear regression and multiple linear regression were also used to find out the relationship between treatments and the different outcomes considered in this study, adjusting for the generated propensity score. The odds ratios (OR) and estimates with the 95% confidence intervals (CI) were reported for the associations. We considered a *P* value of <.05 as statistically significant and used SAS version 9.4 for all statistical analyses.

### 2.8 Ethical consideration

The study was approved by King Abdullah International Medical Research Center Institutional Review Board, Riyadh, Saudi Arabia. Participants’ confidentiality was strictly observed throughout the study by using anonymous unique serial number for each subject and restricting data only to the investigators. Informed consent was not required due to the research's method as per the policy of the governmental and local research center.

## 3 RESULTS

### 3.1 Patient characteristics

The study included 387 patients, of which 49 (12.6%) had received Esomeprazole 20 mg during the study period. Table 1 depicts the baseline characteristics between Esomeprazole 20 mg and Esomeprazole 40 mg treatment groups. Patients who received Esomeprazole 20 mg were older, more likely to be males, had lower BMI, lower Bilirubin, lower requirement of FIO_2_. When adjusted for propensity score using APACHE II score, all these differences became insignificant. Additionally, eGFR, serum creatinine, previous C. difficle (Within 6 months of ICU admission), INR baseline, platelets baseline, PaO2/FiO2 ratio, and Vasoactive Inotropic Score (Within 24 hours of ICU admission) were not significant. On the other hand, patients in esomeprazole 40 mg group have a lower GCS baseline and received deeper sedation within 24 hours of ICU admission.

**TABLE 1:** Baseline characteristics of the Esomeprazole 20 mg and Esomeprazole 40 mg treatment groups

	Esomeprazole20 mg (N = 49)	Esomeprazole 40 mg (N = 338)	*P* value	Estimates (SE)/OR	95%CI	PS adjusted *P*‐value
Age (years) mean ± SD	57.93 (25.36)	57.35 (20.42)	0.7663^	−0.848 (3.425)	(−7.561, 5.864)	0.8044^$*^
BMI (kg/m^2^) mean ± SD	25.77 (8.26)	31.05 (19.35)	0.0013^	4.972 (2.843)	(−0.600, 10.544)	0.0803^$*^
Gender						
(Male)	23 (52.3)	211 (67.4)	0.0478^^	2.24	(1.197, 4.192)	0.0117^$^
CVA (Stroke), n (%)	7 (16.3)	44 (14.1)	0.6966^^	0.841	(0.349, 2.023)	0.6983^$^
HTN, n (%)	23 (53.5)	166 (53.0)	0.9555^^	0.94	(0.493, 1.792)	0.85^$^
Asthma, n (%)	3 (7.0)	21 (6.7)	0.6542**	1.376	(0.309, 6.135)	0.6753^$^
DM, n (%)	21 (48.8)	156 (49.8)	0.9018^^	1.091	(0.579, 2.057)	0.7866^$^
Chronic kidney disease (CKD), n (%)	8 (18.6)	52 (16.6)	0.7436^^	0.867	(0.368, 2.047)	0.7456^$^
Ischemic heart disease (IHD), n (%)	6 (14.0)	43 (13.7)	0.9693^^	1.015	(0.406, 2.538)	0.9742^$^
Atrial fibrillation (AFib), n (%)	4 (9.3)	35 (11.2)	0.9853^^	1.263	(0.425, 3.756)	0.6745^$^
Heart failure (HF), n (%)	6 (14.3)	43 (13.7)	0.9230^^	0.901	(0.354, 2.292)	0.8263^$^
Acute Coronary Syndrome (ACS),n (%)	1 (2.4)	15 (4.8)	0.7048**	1.903	(0.244, 14.867)	0.5396^$^
Dyslipidemia (DLP), n (%)	6 (14.0)	55 (17.6)	0.5549^^	1.183	(0.476, 2.944)	0.7173^$^
Hypothyrodism, n (%)	6 (14.0)	28 (8.9)	0.2753**	0.525	(0.215, 1.283)	0.1575^$^
Cancer, n (%)	3 (7.0)	40 (12.8)	0.2736^^	1.123	(0.418, 3.017)	0.8186^$^
Liver disease (any type), n (%)	3 (7.0)	16 (5.1)	0.7141**	0.709	(0.197, 2.556)	0.5995^$^
Chronic obstructive pulmonary disease (COPD), n (%)	2 (4.7)	28 (9.0)	0.5568**	2.114	(0.486, 9.189)	0.3181^$^
Previous C. difficle (Within 6 months of ICU admission)	1 (2.27)	2 (0.62)	0.3190	1.251	(0.585, 2.676)	0.5636^$^
Estimated glomerular filtration rate (eGFR) (mL/min/1.73m2), mean ± SD	71.48 (52.93)	73.37 (48.91)	0.6802^	3.010 (7.933)	(−12.538, 18.559)	0.7043^$*^
Acute kidney injury	10 (23.26)	95 (30.35)	0.3387	1.251	(0.585, 2.676)	0.5636^$^
Bilirubin (µmol/L), mean ± SD	27.85 (57.02)	28.58 (56.59)	0.0362^	0.613 (9.534)	(−18.074, 19.299)	0.9488^$*^
INR, mean ± SD	1.91 (1.86)	1.58 (1.16)	0.7150^	−0.303 (0.200)	(−0.694, 0.089)	0.1295^$*^
Platelets count (x 109/L), mean ± SD	227.33 (126.07)	221.16 (139.20)	0.4854^	−4.673 (21.576)	(−46.962, 37.616)	0.8285^$*^
aPTT, mean ± SD	42.99 (38.99)	38.29 (23.48)	0.6254^	−4.350 (3.984)	(−12.159, 3.458)	0.2749^$*^
ALT, mean ± SD	151.00 (509.89)	127.08 (388.80)	0.9030^	−26.382 (67.180)	(−158.052, 105.288)	0.6945^$*^
AST, mean ± SD	188.54 (485.58)	198.87 (638.51)	0.8114^	3.776 (101.966)	(−196.074, 203.627)	0.9705^$*^
Albumin (g/L), mean ± SD	29.49 (6.83)	29.64 (5.64)	0.8761*	−0.081 (0.937)	(−1.918, 1.757)	0.9315^$**^
PaO2/FiO_2_ ratio, mean ± SD	236.11 (160.02)	277.18 (180.70)	0.1428^	41.884 (29.732)	(−16.389, 100.157)	0.1589^$*^
FIO_2_ requirement (%)	40.51 (17.85)	43.31 (16.85)	0.0444^	2.355 (1.968)	(−1.502, 6.212)	0.2314^$*^
GCS baseline, mean ± SD	9.50 (4.88)	8.45 (4.48)	0.1624^	−1.499 (0.731)	(−2.932,−0.065)	0.0404^$*^
Lowest RASS, mean ± SD	−2.71 (1.78)	−3.25 (1.34)	0.1181^	−0.629 (0.246)	(−1.111,−0.147)	0.0106^$*^
Glu2 (mmol/L), mean ± SD	12.01 (6.12)	12.57 (5.77)	0.1803^	0.777 (0.655)	(−0.508, 2.061)	0.236^$*^
Chloride (mmol/L), mean ± SD	104.65 (6.97)	106.07 (9.35)	0.1164^	1.544 (1.421)	(−1.241, 4.328)	0.2772^$*^
Lactic acid (mmol/L), mean ± SD	3.41 (2.89)	4.01 (4.23)	0.4526^	0.497 (0.670)	(−0.817, 1.810)	0.4587^$*^
Hematocrit (Hct), mean ± SD	0.34 (0.09)	0.33 (0.08)	0.2858^	−0.014 (0.013)	(−0.040, 0.012)	0.3027^$*^
Vasoacive Inotropic Score (VIS)_24 h, mean ± SD	27.79 (97.79)	55.60 (292.53)	>0.9999^	0.042 (0.136)	(−0.226, 0.309)	0.7589^$*^
BUN (mmol/L), mean ± SD	11.02 (9.42)	11.32 (9.11)	0.9898^	0.449 (1.464)	(−2.420, 3.318)	0.7589^$*^
Bicarbonate (CO_2_), mean ± SD	19.85 (6.27)	19.60 (5.38)	0.4252^	−0.259 (0.868)	(−1.961, 1.442)	0.7653^$*^

Note

Denominator of the percentage is the total number of patients. *T‐Test/^Wilcoxon rank sum test is used to calculate the *P*‐value. ^^Chi‐square test is used to calculate the *P*‐value. $*Propensity score adjusted Generalized linear model is used to calculate estimates and *P*‐value. $**Propensity score adjusted multiple regression model is used to calculate estimates and *P*‐value. $ propensity score adjusted Logistic regression is used to calculate Odds ratio and *P*‐value. **Fisher Exact test is used to calculate the *P*‐value.

Table 2 shows the severity illness baseline between Esomeprazole 20 mg and Esomeprazole 40 mg treatment groups. The severity illness baseline (APACHE II score, SOFA score) and nutrition risk (NUTRIC score) within 24 hours of ICU admission were not statistically significant.

**TABLE 2:** Comparison baseline Severity between Esomeprazole 20 mg and Esomeprazole 40 mg treatment groups

	Esomeprazole 20 mg (N = 49)	Esomeprazole 40 mg (N = 338)	*P* value	Estimates	95%CI	*P*‐value
APACHE II	19.81 (8.10)	19.47 (9.93)	0.4846^	1.093 (1.539)	(−1.923, 4.109)	0.4775^$*^
NUTRIC	4.79 (2.33)	4.48 (2.29)	0.3657^	−0.095 (0.373)	(−0.826, 0.636)	0.7986^$*^
SOFA	7.02 (3.27)	7.32 (3.65)	0.8786^	0.583 (0.573)	(−0.540, 1.705)	0.3091^$*^

Note

*T‐Test/^Wilcoxon rank sum test is used to calculate the *P*‐value. $*Propensity score adjusted Generalized linear model is used to calculate estimates and *P*‐value.

### 3.2 Outcomes

The association between Esomeprazole prophylaxis dose and GI bleeding using multivariate analysis adjusted for propensity score is summarized in Table 2. Compared with esomeprazole 20 mg, esomeprazole 40 mg was not superior in GI bleeding prevention (adjusted OR 2.611, 95% CI 0.343‐20.247, *P* = .356) Table 3. In addition, neither ICU C. difficle (aOR 0.246, 95% CI 0.058‐1.032, *P* = .0552), ICU mortality within 30 days (adjusted OR 0.666, 95% CI 0.333‐1.331, *P* = .249), ICU LOS (aOR 2.515, 95% CI −1.028‐6.059, *P* = .1641), Hospital LOS (aOR 11.155, 95% CI −8.822‐31.132, *P* = .2738), ICU re‐admission within 6 months (aOR 2.029, 95% CI 0.601‐6.848, *P* = .254) RBCs transfusion (Estimates(STD) 1.845 (1.269), 95% CI −0.642‐4.333, *P* = .1459), nor platelets transfusion (Estimates(STD) −6.149 (4.892), 95% CI −15.738‐3.440, *P* = .2088) were significant. On the other hand, esomeprazole 40 mg was statistical associated with Enterobacteriaceae (aOR 2.011, 95% CI 1.005‐4.022, *P* = .048), Pneumonia (aOR 2.563, 95% CI 1.192‐5.510, *P* = .0159) and longer mechanical ventilation duration (Estimates(STD) 3.297 (1.411), 95% CI 0.532 −6.061, *P* = .0194).

**TABLE 3:** Outcomes of the Esomeprazole 20 mg and Esomeprazole 40 mg treatment groups

	Esomeprazole 20 mg (N = 49)	Esomeprazole 40 mg (N = 338)	*P* value	Risk aOR	95%CI	*P* value
GI Bleeding, n (%)	1 (2.04)	20 (5.92)	0.4955**	2.611	0.343, 20.247	0.3562^$^
ICU mortality within 30 days, n (%)	18 (40)	109 (33.33)	0.3766^^	0.666	0.333, 1.331	0.2495^$^
ICU re‐admission within 6 months, n (%)	3 (6.25)	42 (12.46)	0.2100**	2.029	0.601, 6.848	0.2542^$^
Enterobacteriaceae, n (%)	13 (28.89)	150 (46.30)	0.0276^^	2.011	1.005, 4.022	0.0483^$^
ICU C.difficle, n (%)	4 (8.89)	6 (1.84)	0.0230**	0.246	0.058, 1.032	0.0552^$^
Pneumonia, n (%)	11 (28.95)	147 (48.84)	0.0206^^	2.563	1.192, 5.510	0.0159^$^
Bacteremia, n (%)	9 (23.68)	72 (23.92)	0.9743^^	0.953	0.427, 2.127	0.9059^$^
Significant UTI, n (%)	9 (47.37)	54 (50.47)	0.8034^^	1.135	0.407, 3.170	0.8086^$^
Esomeprazole treatment dose, n (%)	2 (4.08)	28 (8.31)	0.4015	1.898	0.434, 8.294	0.3943^$^
Continuous parameters						
RBCs Transfusion (U)	3.6 (6.11)	5.6 (6.51)	0.0098^	1.845 (1.269)	(−0.642, 4.333)	0.1459^$*^
Platelets Transfusion (U)	13.3 (32.86)	7.4 (18.16)	0.3094^	−6.149 (4.892)	(−15.738, 3.440)	0.2088^$*^
Plasma (FFP) transfusion (U)	3.8 (8.31)	3.3 (4.57)	0.2002^	−0.537 (1.137)	(−2.766, 1.692)	0.6369^$*^
Hospital LOS, Median (IQR)	39.4 (53.19)	51.9 (64.38)	0.0355^	11.155 (10.192)	(−8.822, 31.132)	0.2738^$*^
ICU LOS, Median (IQR)	13.1 (12.60)	15.7 (11.36)	0.0171^	2.515 (1.808)	(−1.028, 6.059)	0.1641^$*^
MV duration, Median (IQR)	8.1 (10.05)	10.59 (8.64)	0.0011^	3.297 (1.411)	(0.532, 6.061)	0.0194^$*^

Note

Denominator of the percentage is the total number of patients. *T‐Test/^Wilcoxon rank sum test is used to calculate the *P*‐value. ^^Chi‐square test is used to calculate the *P*‐value. $*propensity score adjusted Generalized linear model is used to calculate estimates and *P*‐value. $**propensity score adjusted multiple regression model is used to calculate estimates and *P*‐value. $ propensity score adjusted Logistic regression is used to calculate Odds ratio and *P*‐value. **Fisher Exact test is used to calculate the *P*‐value.

## 4 DISCUSSION

In the practice, stress ulcer prophylaxis (SUP) continues to be the standard of care in patients admitted to intensive care units (ICU) and Proton pump inhibitors (PPIs), are the most used agents.^5^ Our study aimed to study the efficacy as well as safety between two different regimens of esomeprazole as stress ulcer prophylaxis in a critically ill patient with major risk factors of stress ulceration. Requiring MV longer than 24 hours and/or coagulopathy are two independent risk factors for stress ulceration in critically ill patients. Several studies have been shown the superiority in efficacy and safety of proton pump inhibitors over placebo or H2RA’s as stress ulcer prophylaxis in critically ill patients. For instance, a meta‐analysis that compared the efficacy and safety of PPI’s vs H2RA’s significantly decreased clinically important GI bleeding. Moreover, overt GI bleeding was significantly lower in with PPI’s.^5^, ^9^ Al‐Hazzani, Waleed et al have predefined clinically important bleeding (CIB) as evidence of upper GI bleeding with any of significant hemodynamic changes not explained by other causes, need for transfusion of more than two units of blood, a significant decrease in hemoglobin level, evidence of bleeding on GI endoscopy, or need for surgery to control the bleeding. While overt bleeding was defined as evidence of upper GI bleeding (hematemesis, melena, hematochezia, or coffee‐grounds emesis or aspirate) regardless of other clinical findings.^10^ In our study, we denominated GI bleeding by PPI treatment dose administration accompanied by clear diagnostic documentation or positive endoscopy examination during ICU stay. Initially, enrolled participants for the treatment groups have similar baseline characteristics. Despite the fact that the results of our study indicate an insignificant difference between the two studied PPI doses (Esomeprazole 20 mg and Esomeprazole 40 mg) in terms of superiority, interestingly, it has been shown that the incidence of Enterobacteriaceae infection was greater in the treated group with Esomeprazole 40 mg. In a case‐control study published in 2018, they found that PPI exposure within the previous six months is significantly associated with infection with both extended‐spectrum beta‐lactamase (ESBL)‐producing Enterobacteriaceae ESBL‐ and non‐ESBL‐producing bacteria, while H2A and antacids were not significantly associated with infection.^10^ In line with their hypothesis it is stated that reducing inappropriate use of PPIs may be a novel way to reduce transmission, which might reduce antibiotic use and help control antimicrobial resistance in ICU patient, our result supported their finding as higher doses of PPI was associated with a higher incidence of Enterobacteriaceae infections. An association between PPIs use and C. diff infection (CDI) is at least theoretically rational, several systematic reviews and meta‐analyses have reported conflicting results regarding the association between PPIs use and increased risk of CDI.^11^ An updated meta‐analysis published in 2017 provided evidence that PPI use is associated with an increased risk for the development of C. diff infections. However, the study was limited by a lack of details regarding the dose and duration of PPI. Based on our findings ICU C. diff infection was statistically not significant between Esomeprazole 20 and 40 mg. Contrary with the hypothesized association, David M. Faleck et al found that PPIs did not increase the risk for C. diff infection in the ICU patients regardless of use of the antibiotics.^12^

In terms of RBCs and platelets transfusion requirement, there was no significant difference in both study groups. Nevertheless, patients with Esomeprazole 20 mg have shorter mechanical ventilation duration compared with Esomeprazole 40 mg. No enough studies investigating MV duration among different regimens of esomeprazole. In addition, the NUTRIC score was calculated to determine nutritional risk assessment for ICU patients and adjusted for both groups to eliminate confounding variables. In parallel, with many studies that show a high incidence of ventilator‐associated pneumonia (VAP) in patients treated with PPI, our study shows that esomeprazole 40 mg was statistically associated with, Pneumonia compared with esomeprazole 20 mg.^13^

This study was considered first of its kind by comparing two dosing regimens of esomeprazole as SUP. Suggesting that lower doses of esomeprazole (ie 20 mg once daily) as SUP provides appropriate therapeutic efficacy in preventing stress ulceration with lower consequences and costs in critically ill patients. Our study is limited by small sample size and retrospective design. Furthermore, some of the patients were started empiric treatment of esomeprazole without confirming the diagnosis with GI bleeding or incomplete documentation, which would complicate data retrieving.

## 5 CONCLUSION

In conclusion, our study found that Esomeprazole 40 was not superior to Esomeprazole 20 in terms of preventing GI bleeding in critically ill patients. On the other hand, pneumonia, Enterobacteriaceae infections, and MV duration were significantly higher with Esomeprazole 40. These data confirm the need for randomized controlled trials with a larger sample size to clarify and confirm our study findings.
